# Impact of serum magnesium and bone mineral density on systemic fractures in chronic hemodialysis patients

**DOI:** 10.1371/journal.pone.0251912

**Published:** 2021-05-20

**Authors:** Mayuko Hori, Kaoru Yasuda, Hiroshi Takahashi, Chikao Yamazaki, Kunio Morozumi, Shoichi Maruyama

**Affiliations:** 1 Department of Nephrology, Masuko Memorial Hospital, Nakamura-ku, Nagoya, Aichi, Japan; 2 Department of Nephrology, Fujita Health University School of Medicine, Toyoake, Aichi, Japan; 3 Masuko Clinic Subaru, Nakamura-ku, Nagoya, Aichi, Japan; 4 Department of Nephrology, Nagoya University Graduate School of Medicine, Showa-ku, Nagoya, Aichi, Japan; Medical College of Wisconsin, UNITED STATES

## Abstract

**Introduction:**

Bone mineral density (BMD) measured with dual-energy X-ray absorptiometry (DXA) can be used to predict fractures, but its clinical utility has not been fully established in chronic kidney disease (CKD) patients. Magnesium is an essential trace element. Although magnesium is associated with the risk of fractures in non-CKD populations, the relationship is unknown in CKD patients.

**Methods:**

BMD and serum magnesium levels were measured in 358 stable outpatients undergoing maintenance hemodialysis therapy. The primary outcome was fragility fracture. Patients were divided into groups according to the median level of magnesium and the normal threshold value of lumbar spine BMD.

**Results:**

During the median follow-up period of 36 months, 36 (10.0%) fractures occurred. The cumulative incidence rates of fractures were 17.6% and 5.2% [adjusted hazard ratio (aHR) 2.31, 95% confidence interval (CI) 1.03–5.17, P = 0.030] in the lower (<2.6 mg/dL) and higher (≥2.6 mg/dL) magnesium (Mg) groups, respectively, and 21.2% and 7.3% (aHR 2.59, 95% CI 1.09–6.16, P = 0.027) in the low- and high-BMD groups, respectively. The lower-Mg and low-BMD group had a 9.21-fold higher risk of fractures (95% CI; 2.35–47.00; P = 0.0010) than the higher-Mg and high-BMD group. Furthermore, adding both magnesium levels and lumbar spine BMD levels to the established risk factors significantly improved the prediction of fractures (C-index: 0.784 to 0.830, p = 0.041).

**Discussion/Conclusions:**

The combination of serum magnesium and lumbar spine BMD can be used for fracture risk stratification and synergistically improves the prediction of fractures in CKD patients.

## Introduction

Fractures are a major source of morbidity and mortality in patients undergoing hemodialysis [1,2]. The risk of any fracture is higher in patients undergoing dialysis than in the general population [3–7]. Although low bone mineral density (BMD) is a strong risk factor for fractures among healthy men and women, the clinical utility of measuring BMD by dual-energy X-ray absorptiometry (DXA) might be limited in patients with chronic kidney disease mineral bone disorder (CKD-MBD), particularly those with stages 5 and 5D disease, because there are alterations in the bone microarchitecture that are not observable on DXA imaging [8].

Numerous factors, such as older age, female sex, low body mass index and muscle weakness, are known to increase the fracture risk in patients undergoing dialysis [5,9–11]. However, investigations of the effects of hormonal factors and nutrition on bone health have mostly focused on calcium and vitamin D deficiencies and altered parathyroid hormone (PTH) levels [12].

Magnesium (Mg) is an essential trace element that plays a key role in cellular processes and is an important component of bone; 67% of the magnesium in the body is found in bone tissue [13]. Several animal studies have shown that magnesium deficiency is associated with decreased osteoblast function, increased osteoclast function and skeletal fragility [14,15]. A recent meta-analysis of 12 studies reported that a higher magnesium intake level was positively associated with the BMD of the femoral neck and total hip [16]. Some cohort studies in non-CKD populations have reported that lower serum magnesium levels were associated with an increased risk of fractures, including hip fractures [17,18]. Although magnesium might affect the risk of fractures, little is known about the relationship between magnesium and the risk of fractures in patients with CKD. Recently, one cohort study of patients undergoing hemodialysis found that lower serum magnesium levels were associated with a higher risk of hip fractures [19]. In this study, our objective was to assess the association of serum magnesium and BMD with the risk of incident fractures and to determine whether the prediction of the risk of fractures in patients undergoing hemodialysis based on the combination of magnesium level and lumbar spine BMD is more accurate than the prediction based on either variable alone.

## Materials and methods

### Study population

A total of 358 patients who had undergone hemodialysis for at least 2 months and had baseline measurements of serum magnesium and BMD at Masuko Memorial Hospital between May 2016 and November 2017 were enrolled. Patients undergoing combination therapy with peritoneal dialysis were excluded. This study was performed in accordance with the Helsinki Declaration and was approved by Masuko Memorial Hospital Ethics Committee (Ethics approval number: MR2-19). Due to the retrospective nature of the study, written informed consent was waived. All data were anonymized before analysis and the data range was from May 2016 to July 2020.

### Baseline covariates

The following data were collected: demographics (age, sex, body mass index, hemodialysis vintage, duration of hemodialysis treatment); laboratory measurements (predialysis albumin, urea nitrogen, calcium, phosphate, magnesium, C-reactive protein [CRP], hemoglobin, alkaline phosphatase [ALP], and intact parathyroid hormone [iPTH]; prescriptions (phosphate binders, cinacalcet hydrochloride, active vitamin D analogues and proton pump inhibitors); and past history of parathyroidectomy, cardiovascular diseases (myocardial infarction, cerebral infarction, and cerebral hemorrhage) and incident fractures. For determination of biochemical parameters, serum was obtained immediately before the first weekly dialysis treatment at baseline. The dialysate magnesium concentration was 1.2 mg/dL.

### Measurements of BMD

For the assessment of BMD, we used dual-energy X-ray absorptiometry (DXA) because it is the most widely used tool for the assessment of bone mass and fracture risk in the general population. BMD of the lumbar spine (L2-L4), 1/3 distal radius and total hip is reported in g/cm^2^ or the T-score (calculated using the mean and SD for Japanese young adults). DXA was performed with the Lunar iDXA system (GE Health Care Japan, Tokyo, Japan). According to the World Health Organization classification system, osteoporosis was defined as a BMD T-score of -2.5 or less, osteopenia as a T-score between -1 and -2.5, and normal BMD as a T-score of -1 or more.

### Follow-up period

The follow-up period was censored on July 2020. Patients were followed for up to 3 years. The study endpoint was defined as any type of new fragility fracture. We defined fragility fractures as low energy fractures occurring in falls from standing height or less. We identified the asymptomatic fractures with annual whole-body skeletal X-ray survey and detected the symptomatic fractures diagnosed with radiological examinations from the hospital chart.

### Statistical analysis

Baseline characteristics are presented as the means (standard deviations) or medians (interquartile range) for continuous variables and percentages for categorical variables. For continuous variables, the differences between two groups were evaluated using the Mann-Whitney U test or Student’s t-test, and the differences among three or more groups were assessed with ANOVA or the Kruskal-Wallis test. For categorical variables, the chi-squared test was used. Kaplan–Meier cumulative event curves were constructed to describe the frequency of events among the groups with various risk factors and were compared with the log-rank test. The cut-off level for BMD was defined based on the normal reference range (T-score ≥ -1.0) in the system established by the World Health Organization, and the cut-off level for serum magnesium level was defined based on the median value. Hazard ratios and 95% confidence intervals (CIs) were calculated using Cox regression models to estimate the relationships between the variables and the outcome. To identify the independent predictors of the endpoints, all baseline variables with P < 0.05 in univariate analysis were entered into a multivariate model. We also calculated the C-index, net reclassification improvement (NRI) and integrated discrimination improvement (IDI) to assess whether the predictive ability for fractures was improved after the addition of Mg, BMD or both to a baseline model with established risk factors. The C-index was defined as the area under receiver-operating characteristic curves between individual predictive probabilities for fracture and the incidence of fracture, and was compared among each predicting model [20]. The NRI relatively indicates how many patients improve their predicted probabilities for fracture, while the IDI represents the average improvement in predicted probabilities for fracture after adding variables into the baseline model [21].

All reported P values were 2-sided, and P values < 0.05 were considered statistically significant. All statistical analyses were performed using JMP^®^ 11 (SAS Institute Inc., Cary, NC, USA) and R version 3.4.1.

## Results

### Patient characteristics

The baseline characteristics are shown in Tables 1 and 2. During a median (interquartile range) follow-up of 3.0 (2.8–3.0) years, 36 incident fractures (hip fractures: 10, vertebral fractures: 18, other fractures: 8) occurred.

**Table 1:** Baseline characteristics of the groups stratified by serum magnesium levels.

Groups based on the serum Mg level	Total n = 358	Lower Mg (≤ 2.5 mg/dL) n = 190	Higher Mg (≥ 2.6 mg/dL) n = 168	P-value
Demographics				
Age (yrs)	65.6 ± 14.3	69.1 ± 14.1	61.6 ± 13.4	< 0.0001
Sex (%), male	74.0	74.6	73.1	0.74
BMI (kg/m^2^)	21.4 ± 4.2	21.3 ± 4.6	21.6 ± 3.7	0.60
DM (%)	44.1	43.2	45.2	0.69
HD vintage (yrs)	6.2 (1.8, 12.0)	5.6 (1.5, 11.6)	6.7 (2.1, 12.5)	0.49
HD duration (hours/week)	13.5 ± 3.4	13.3 ± 3.2	13.6 ± 3.5	036
Laboratory data				
BUN (mg/dL)	56.4 ± 12.8	54.4 ± 13.1	58.7 ± 12.0	0.0014
Adj. Calcium (mg/dL)	9.0 ± 0.5	9.0 ± 0.6	9.0 ± 0.5	0.71
Phosphate (mg/dL)	5.2 ± 1.2	5.0 ± 1.2	5.4 ± 1.1	0.0013
Magnesium (mg/dL)	2.5 ± 0.3	2.2 ± 0.2	2.8 ± 0.2	< 0.0001
CRP (mg/dl)	0.13 (0.05, 0.32)	0.19 (0.06, 0.46)	0.09 (0.05, 0.21)	< 0.0001
Alb (g/dL)	3.5 ± 0.3	3.4 ± 0.4	3.7 ± 0.2	< 0.0001
Hb (g/dL)	11.1 ± 1.2	10.9 ± 1.2	11.4 ± 1.1	< 0.0001
ALP (IU/L)	267 ± 133	280 ± 125	252 ± 140	0.047
iPTH (pg/mL)	142 (92, 214)	141 (99, 212)	144 (86, 219)	0.85
Total hip DXA BMD (g/cm^2^)	0.75 ± 0.17	0.75 ± 0.18	0.76 ± 0.15	0.49
L2-L4 DXA BMD (g/cm^2^)	1.12 ± 0.24	1.13 ± 0.25	1.11 ± 0.23	0.36
1/3 Radius DXA BMD (g/cm^2^)	0.74 ± 0.19	0.72 ± 0.20	0.76 ± 0.18	0.10
Past history				
CVD (%)	31.8	38.9	23.8	0.0022
Parathyoidectomy (%)	3.0	3.2	3.0	0.9
Prevalent fracture (%)	13.4	19.5	6.5	0.0003
Medication				
CaCO3 (%)	47.4	41.1	54.8	0.0095
Phophate binders (%)	55.5	49.5	62.5	0.013
Vitamin D (%)	75.1	75.8	74.4	0.76
Cinacalcet (%)	28.4	28.4	28.6	0.9
PPI (%)	55.8	61.5	49.4	0.020

Data are expressed as the mean ± SD or median (interquartile range).

BMI, body mass index; DM, diabetes mellitus; BUN, blood urea nitrogen; Adj. Calcium, albumin-adjusted calcium; CRP, C-reactive protein; Alb, albumin; Hb, hemoglobin; ALP, alkaline phosphatase; iPTH, intact parathyroid hormone; CVD, cardiovascular disease.

**Table 2:** Baseline Characteristics of the groups according to L2-L4 BMD (T-score).

Groups based on L2-L4 BMD	Low L2-L4 BMD n = 109	High L2-L4 BMD n = 246	P-value
Demographics			
Age (yrs)	69.2 ± 15.1	63.9 ± 13.6	0.0010
Sex (%) male	42.2	88.6	< 0.0001
BMI (kg/m^2^)	18.9 ± 2.5	22.6 ± 4.3	< 0.0001
DM (%)	32.1	49.5	0.0022
HD vintage (yrs)	7.8 (3.4, 13.6)	4.9 (1.4, 11.2)	0.0019
HD duration (hours/week)	12.8 ± 2.9	13.7 ± 3.6	0.0022
Laboratory data			
BUN (mg/dL)	56.6 ± 13.2	56.4 ± 12.6	0.9
Adj. Calcium (mg/dL)	9.1 ± 0.6	9.0 ± 0.5	0.20
Phosphate (mg/dL)	5.1 ± 1.1	5.2 ± 1.2	0.21
Magnesium (mg/dL)	2.5 ± 0.3	2.5 ± 0.3	0.18
CRP (mg/dl)	0.09 (0.05, 0.27)	0.14 (0.06, 0.33)	0.077
Alb (g/dL)	3.5 ± 0.4	3.6 ± 0.3	0.080
Hb (g/dL)	11.1 ± 1.0	11.1 ± 1.2	0.9
ALP (IU/L)	302 ± 164	251 ± 111	0.0007
iPTH (pg/mL)	144 (98, 224)	144 (91, 213)	0.45
Total hip DXA BMD (g/cm^2^)	0.57 ± 0.17	0.82 ± 0.14	< 0.0001
L2-L4 DXA BMD (g/cm^2^)	0.85 ± 0.09	1.24 ± 0.19	< 0.0001
1/3 Radius DXA BMD (g/cm^2^)	0.61 ± 0.12	0.82 ± 0.14	< 0.0001
Past history			
CVD (%)	28.4	33.7	0.32
Parathyoidectomy (%)	4.5	2.4	0.28
Prevalent fracture (%)	18.3	10.9	0.058
Medication			
CaCO3 (%)	42.2	50.0	0.17
Phosphate binders (%)	56.8	55.6	0.83
Vitamin D (%)	71.5	76.8	0.28
Cinacalcet (%)	33.9	26.4	0.14
PPI (%)	62.3	52.4	0.082

Data are expressed as the mean ± SD or median (interquartile range).

BMI, body mass index; DM, diabetes mellitus; BUN, blood urea nitrogen; Adj. Calcium, albumin-adjusted calcium; CRP, C-reactive protein; Alb, albumin; Hb, hemoglobin; ALP, alkaline phosphatase; iPTH, intact parathyroid hormone; CVD, cardiovascular disease; Low L2-L4 BMD, L2-L4 BMD T-score < -1.0; High L2-L4 BMD, L2-L4 BMD T-score ≥ -1.0.

### Predictive value of serum magnesium and BMD for fractures

The multivariate Cox proportional hazards analysis identified serum magnesium level (adjusted HR 0.29; 95% CI 0.10–0.83, P = 0.021) and baseline L2-L4 BMD per 0.1 g/cm^2^ (adjusted HR 0.70; 95% CI 0.52–0.93, P = 0.011) as independent predictors of incident fractures after adjustment for age, body mass index, hemodialysis duration, serum magnesium level, past incident fracture, use of phosphate binders, total hip DXA BMD per 0.1 g/cm^2^, L2-L4 DXA BMD per 0.1 g/cm^2^ and distal 1/3 radius DXA BMD per 0.1 g/cm^2^ as covariates with P values < 0.05 in univariate analysis (Table 3). The patients were divided into two groups based on the median serum Mg level, and no significant differences were observed between the lower-Mg group and the higher-Mg group for total hip DXA BMD, L2-L4 DXA BMD or 1/3 radius DXA BMD (total hip DXA: 0.75 ± 0.18 g/cm^2^ versus 0.76 ± 0.15 g/cm^2^; P = 0.49, L2-L4 DXA: 1.13 ± 0.25 g/cm^2^ versus 1.11 ± 0.23 g/cm^2^; P = 0.36, 1/3 radius DXA: 0.72 ± 0.20 g/cm^2^ versus 0.76 ± 0.18 g/cm^2^; P = 0.10). The patients were divided into two groups based on the normal threshold value of L2-L4 DXA BMD, and there was no significant difference in serum magnesium levels between the low-BMD group and the high-BMD group (2.5 ± 0.3 mg/dL versus 2.5 ± 0.3 mg/dL; P = 0.18). There was no significant difference in iPTH values between the lower- and higher-Mg groups (141 (99, 212) versus 144 (86, 219); P = 0.85) (Tables 1 and 2).

**Table 3:** Cox proportional hazard analysis of the associations of baseline variables with the risk of any fracture.

Variable	Univariate		Multivariate	
	HR (95% CI)	P-value	HR (95% CI)	P-value
Age (yrs)	1.09 (1.06–1.13)	< 0.0001	1.07 (1.03–1.12)	< 0.0001
Sex, male	0.64 (0.33–1.33)	0.23		
BMI (kg/m^2^)	0.89 (0.80–0.98)	0.018	1.03 (0.93–1.12)	0.42
DM	1.30 (0.67–2.51)	0.43		
HD vintage (yr)	1.01 (0.96–1.05)	0.63		
HD duration (hours/week)	0.74 (0.56–0.91)	0.0011	0.92 (0.72–1.10)	0.45
BUN, mg/dL	0.99 (0.97–1.02)	0.87		
Adj. Ca, mg/dL	0.92 (0.52–1.58)	0.78		
P, mg/dL	0.95 (0.71–1.24)	0.73		
Mg, mg/dL	0.18 (0.072–0.48)	0.0006	0.29 (0.10–0.83)	0.021
CRP, mg/dL	1.22 (0.69–1.72)	0.41		
Alb, g/dL	0.45 (0.22–1.02)	0.058		
Hb, g/dL	0.87 (0.65–1.15)	0.33		
ALP, IU/L	1.00 (0.99–1.00)	0.52		
iPTH, pg/mL	1.00 (0.99–1.00)	0.88		
Past history of CVD	1.14 (0.55–2.24)	0.70		
Parathyroidectomy	0.76 (0.043–3.54)	0.79		
Prevalent fracture	7.38 (3.76–14.27)	< 0.0001	3.82 (1.78–8.02)	0.0008
CaCO3	0.70 (0.35–1.35)	0.30		
Phosphate binders	0.44 (0.22–0.86)	0.017	0.58 (0.28–1.17)	0.12
Vitamin D	1.10 (0.52–2.60)	0.79		
Cinacalcet	0.91 (0.41–1.83)	0.80		
PPI	1.89 (0.95–4.00)	0.068		
Total hip DXA BMD (per 0.1 g/cm^2^)	0.72 (0.59–0.89)	0.0026	1.21 (0.84–1.73)	0.29
L2-L4 DXA BMD (per 0.1 g/cm^2^)	0.77 (0.65–0.90)	0.0009	0.70 (0.52–0.93)	0.011
Distal 1/3 Radius DXA BMD (per 0.1 g/cm^2^)	0.73 (0.61–0.86)	0.0002	1.12 (0.83–1.52)	0.43

CI, confidence interval; HR, hazard ratio.

**Fig 1:**
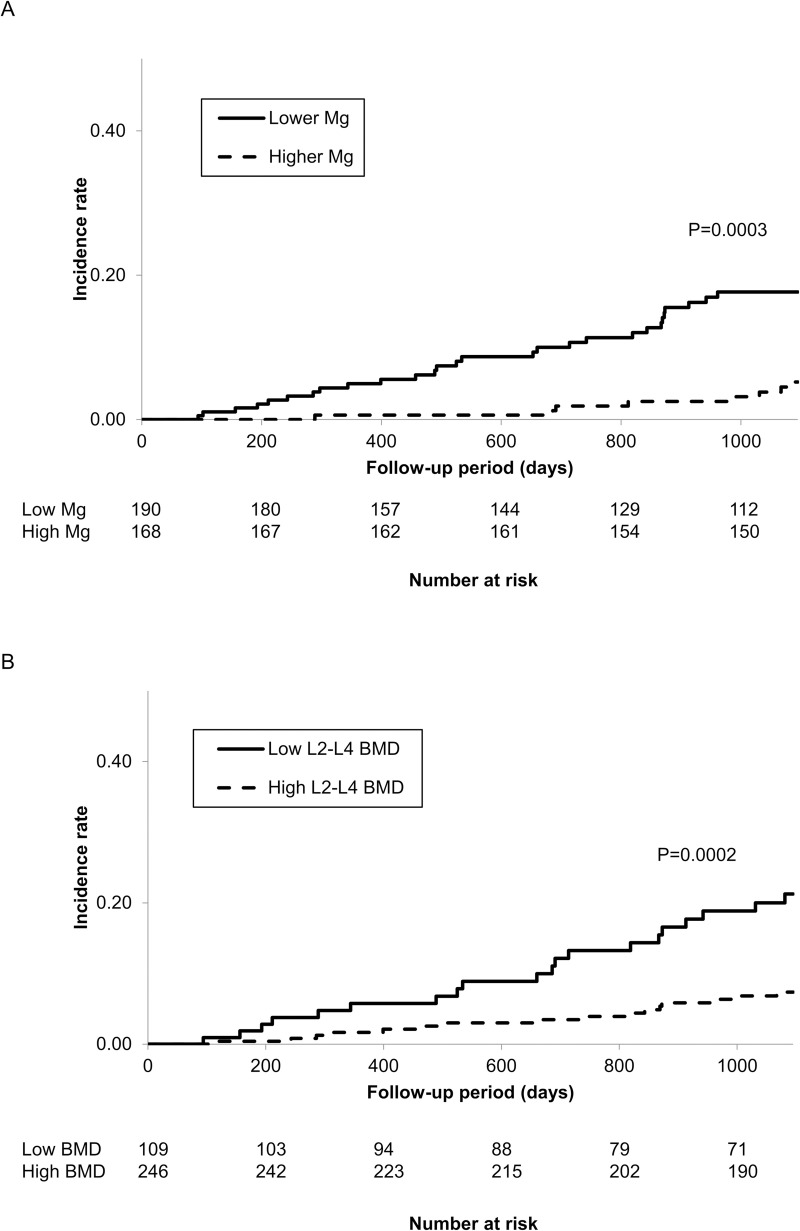
Kaplan-Meier cumulative incidence curves for fractures according to the groups. (A) Stratified by the magnesium level. (B) Stratified by L2-L4 BMD.

The 3-year cumulative incidence rate of fractures was significantly higher in the lower Mg group than in the higher Mg group (17.6% vs. 5.2%, adjusted HR 2.31; 95% CI 1.03–5.17, P = 0.030) and was higher in the low-BMD group than in the high-BMD group (21.2% vs. 7.3%, adjusted HR 2.59; 95% CI 1.09–6.16, P = 0.027) (shown in Fig 1 and Table 4).

**Table 4:** Cox proportional hazard analysis of associations of the Mg level and BMD with the risk of total incident fractures after transforming the Mg levels and BMD into binary categories using the median value or official cut-off value.

Variables	Unadjusted		Adjusted	
	HR (95% CI)	P-value	HR (95% CI)	P-value
Lower Mg (≤ 2.5 mg/dL)	3.91 (1.86–9.22)	0.0002	2.31 (1.03–5.17)	0.030
Total hip BMD T-score < -1.0	3.14 (1.12–13.10)	0.026	1.49 (0.38–5.87)	0.55
L2-L4 BMD T-score < -1.0	3.13 (1.62–6.12)	0.0007	2.59 (1.09–6.16)	0.027
1/3 Radius BMD T-score < -1.0	2.14 (1.04–4.82)	0.036	0.57 (0.21–1.52)	0.27

Adjusted for age, BMI, HD duration, low serum Mg level (≤ 2.5 mg/dL), past incident fracture, use of phosphate binders, total hip BMD T-score < -1.0, L2-L4 BMD T-score < -1.0 and distal 1/3 radius BMD T-score < -1.0, which were the covariates with P<0.05 in the univariate analysis, as shown in Table 3.

### Combined predictive value of Mg and BMD

**Fig 2:**
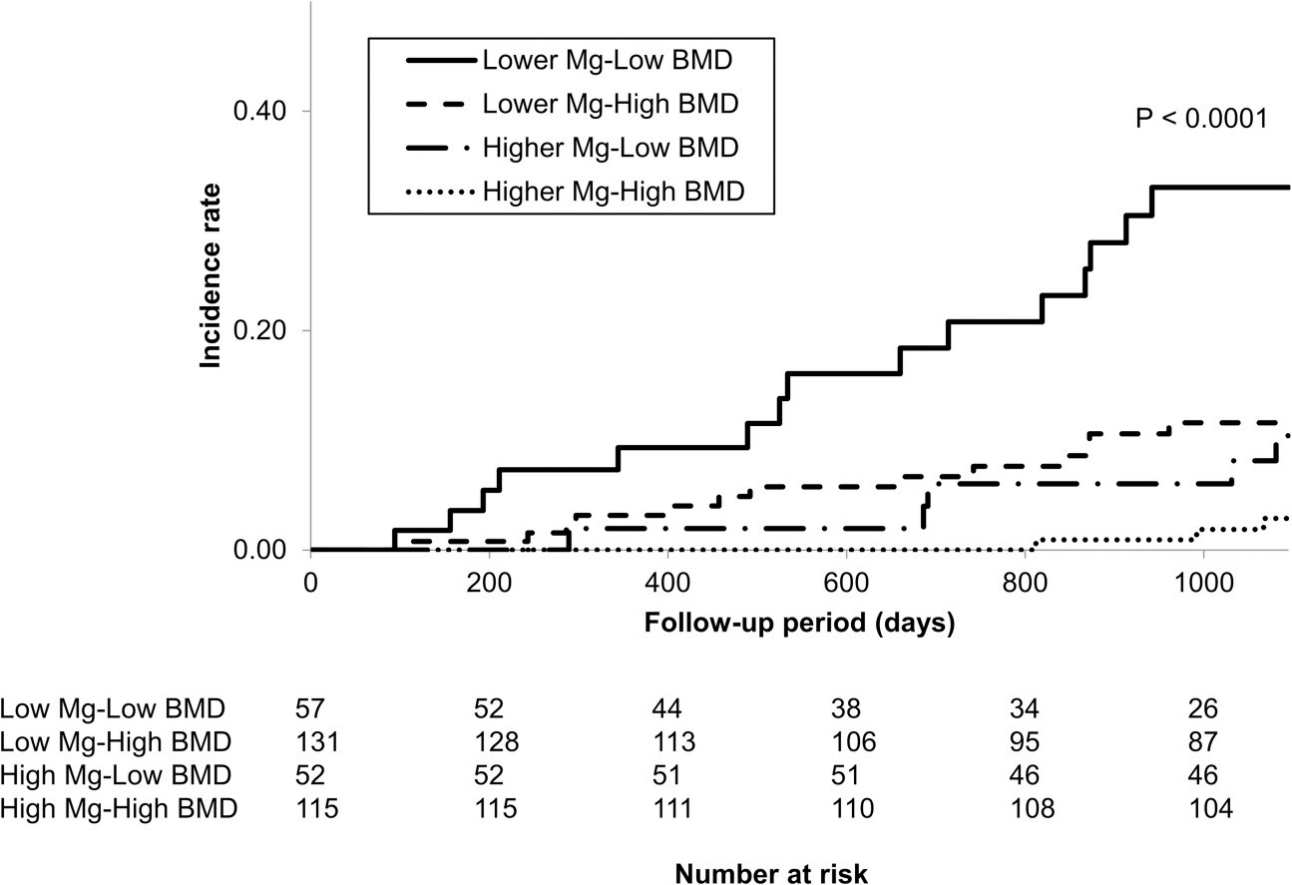
Kaplan-Meier cumulative incidence curves for fractures stratified by both the magnesium level and L2-L4 BMD.

The 3-year cumulative incidence rates of fracture were 2.8%, 10.4%, 11.6% and 33.0% in the higher-Mg and high-BMD group, higher-Mg and low-BMD group, lower-Mg and high-BMD group and lower-Mg and low-BMD group, respectively (P < 0.0001, shown in Fig 2 and Table 5). Considering the higher-Mg and high-BMD group as the reference, the adjusted HRs (95% CIs) for fractures in the lower-Mg and low-BMD group and the higher-Mg and low-BMD group were 9.21 (2.35–47.00; P = 0.0010) and 5.09 (1.01–29.63; P = 0.048), respectively (Table 6).

**Table 5:** Baseline characteristics according to the groups stratified by both the serum Mg level and BMD.

Groups according to the serum Mg level and L2-L4 BMD level	Lower Mg-Low BMD n = 57	Higher Mg-Low BMD n = 52	Lower Mg-High BMD n = 131	Higher Mg-High BMD n = 115	P-value
Demographics					
Age (yrs)	76.0 ± 11.0	61.8 ± 15.6	66.0 ± 14.3	61.4 ± 12.2	< 0.0001
Sex (%), male	38.5	46.1	91.6	85.2	< 0.0001
BMI (kg/m^2^)	18.6 ± 2.4	19.2 ± 2.6	22.5 ± 4.8	22.6 ± 3.7	< 0.0001
DM (%)	33.3	30.7	48.0	51.3	0.021
HD vintage (yr)	7.2 (3.4, 14.0)	7.9 (3.2, 13.1)	4.7 (1.4, 9.9)	5.7 (1.4, 12.5)	0.012
HD duration (hours/week)	12.6 ± 2.7	13.1 ± 3.2	13.6 ± 3.5	13.9 ± 3.7	0.10
Laboratory data					
BUN (mg/dL)	53.7 ± 14.9	59.7 ± 10.4	54.7 ± 12.4	58.3 ± 12.7	0.014
Adj. Calcium (mg/dL)	9.1 ± 0.5	9.0 ± 0.6	9.0 ± 0.5	9.0 ± 0.5	0.51
Phosphate (mg/dL)	4.9 ± 1.2	5.3 ± 0.9	5.0 ± 1.2	5.5 ± 1.3	0.0096
Magnesium (mg/dL)	2.2 ± 0.2	2.8 ± 0.2	2.2 ± 0.2	2.7 ± 0.1	< 0.0001
CRP (mg/dl)	0.17 (0.05, 0.45)	0.06 (0.05, 0.19)	0.19 (0.07, 0.47)	0.10 (0.05, 0.23)	0.0002
Alb (g/dL)	3.3 ± 0.4	3.7 ± 0.3	3.5 ± 0.3	3.7 ± 0.2	< 0.0001
Hb (g/dL)	10.9 ± 1.1	11.3 ± 0.9	10.8 ± 1.2	11.4 ± 1.2	0.0003
ALP (IU/L)	309 ± 119	294 ± 204	265 ± 122	234 ± 95	0.0015
iPTH (pg/mL)	138 (100, 214)	165 (95, 232)	149 (99, 213)	140 (82, 213)	0.65
Total hip DXA BMD (g/cm^2^)	0.59 ± 0.13	0.62 ± 0.11	0.82 ± 0.15	0.82 ± 0.13	< 0.0001
L2-L4 DXA BMD (g/cm^2^)	0.84 ± 0.10	0.87 ± 0.08	1.26 ± 0.18	1.22 ± 0.20	< 0.0001
1/3 radius DXA BMD (g/cm^2^)	0.53 ± 0.16	0.61 ± 0.18	0.82 ± 0.14	0.83 ± 0.14	< 0.0001
Past history					
CVD (%)	35.0	21.1	41.2	25.2	0.014
Parathyoidectomy (%)	3.5	5.7	3.0	1.7	0.57
Prevalent fracture (%)	28.0	7.6	15.2	6.0	0.0005
Medication					
CaCO3 (%)	26.3	59.6	48.0	52.1	0.0025
Phosphate binders (%)	49.1	65.3	50.3	61.7	0.10
Vitamin D (%)	71.9	71.1	77.8	75.6	0.73
Cinacalcet (%)	33.3	34.6	26.7	26.0	0.54
PPI (%)	71.9	51.9	56.4	47.8	0.025

Low Mg, serum Mg ≤ 2.5 mg/dL; High Mg, serum Mg ≥ 2.6 mg/dL; Low L2-L4 BMD, L2-L4 BMD T-score < -1.0; High L2-L4 BMD, L2-L4 BMD T-score ≥ -1.0.

**Table 6:** Adjusted HRs for fractures in the groups stratified by both the serum Mg level and lumbar spine BMD.

	Unadjusted		Adjusted	
	HR (95% CI)	P-value	HR (95% CI)	P-value
Higher Mg and High L2-L4 BMD	Reference	‐	Reference	‐
Lower Mg and High L2-L4 BMD	4.59 (1.47–20.02)	0.0068	2.78 (0.86–12.37)	0.088
Higher Mg and Low L2-L4 BMD	3.80 (0.93–18.55)	0.062	5.09 (1.01–29.63)	0.048
Lower Mg and Low L2-L4 BMD	14.86 (4.86–64.25)	< 0.0001	9.21 (2.35–47.00)	0.0010

Adjusted for age, BMI, HD duration, past incident fracture, use of phosphate binders, total hip DXA BMD and 1/3 radius DXA BMD (significant variables in univariate Cox regression analysis for fractures).

High Mg, serum Mg level ≥ 2.6 mg/dL; Low Mg, serum Mg level ≤ 2.5 mg/dL.

Low L2-L4 BMD, L2-L4 BMD T-score < -1.0; High L2-L4 BMD, L2-L4 BMD T-score ≥ -1.0.

Regarding the discriminatory ability of the model with regard to the prediction of fractures, the addition of both magnesium levels and lumbar spine BMD levels to the prediction model based on established risk factors with P < 0.05 in univariate analysis, namely, age, BMI, hemodialysis duration, past incident fracture, use of phosphate binders, total hip DXA BMD and 1/3 radius DXA BMD, improved the C-index (0.784 to 0.830, P = 0.041), NRI (0.600, P = 0.00044) and IDI (0.041, P = 0.010) significantly more than each alone (Table 7).

**Table 7:** Discriminatory ability of each prediction model for any type of incident fracture based on the C-index, Net Reclassification Improvement (NRI), and Integrated Discrimination Improvement (IDI).

Variables	C-index (95% CI)	P-value	NRI	P	IDI	P-value
Established risk factors	0.784 (0.699–0.869)	Reference		Reference		Reference
Established risk factors + serum Mg	0.789 (0.705–0.873)	0.49	0.174	0.16	0.001	0.31
Established risk factors + L2-L4 BMD	0.825 (0.750–0.899)	0.076	0.497	0.0028**	0.039	0.012*
Established risk factors + serum Mg + L2-L4 BMD	0.830 (0.759–0.901)	0.041*	0.600	0.00044**	0.041	0.010*

Established risk factors include age, BMI, HD duration, past incident fracture, use of phosphate binders, total hip DXA BMD and 1//3 radius DXA BMD as covariates with P<0.05 in the univariate analysis.

## Discussion/Conclusion

In the present study, we demonstrated that a lower serum magnesium level and a lower lumbar spine BMD can be used to predict the incidence of fractures; furthermore, the combination of these variables can be used to stratify patients according to fracture risk and improve the prediction of fractures in patients undergoing hemodialysis. To our knowledge, this is the first study to show the combined predictive value of the serum magnesium level and BMD for the occurrence of fractures.

The usefulness of DXA to classify fracture risk in CKD patients has been controversial because the pathogenesis of renal osteodystrophy is not uniform and may be the result of multiple causes, and BMD measured by DXA does not provide information about the type of renal osteodystrophy [22]. Some past studies with small sample sizes showed that BMD measured by DXA could not be used to identify the fracture risk in hemodialysis patients [23,24]. Despite these limitations, some recent prospective studies found that BMD measured by DXA could be used to predict fractures in patients with CKD [25,26]. Based on these studies, the 2017 Kidney Disease: Improving Global Outcomes (KDIGO) guidelines suggested the measurement of BMD by DXA to assess the fracture risk in advanced CKD patients [27]. The results of our study confirmed the findings of these prior studies with regard to the association between BMD measured by DXA and the fracture risk in end-stage renal disease (ESRD) patients on hemodialysis. To improve the specificity and sensitivity of BMD measured by DXA for the fracture risk, there has been interest in adding clinical risk factors. In this study, we investigated whether combining the BMD and serum magnesium level could increase the accuracy of the prediction of fractures.

A previous large-scale cohort study showed the relationship between magnesium and the risk of fracture among patients undergoing hemodialysis [19]. The serum magnesium level is considered a practical and easy-to-measure marker of the bone magnesium content because the serum magnesium level has been reported to correlate well with the bone magnesium content [28]. Some experimental studies have identified the effects of magnesium deficiency on bone metabolism. *In vitro* studies have shown that a low extracellular magnesium concentration stimulates osteoclastogenesis and inhibits osteoblast proliferation through the upregulation of inducible nitric oxide synthesis [29,30]. In animal studies, a magnesium-deficient diet has been shown to activate osteoclasts and cause excessive bone mineralization, leading to the impairment of bone strength [14,31,32]. The hydroxyapatite crystal size and perfection in bone were first suggested to contribute to the mechanical strength of bones in the early 1980s [33]. A past animal study showed that newly formed crystals of apatite were larger in magnesium-deficient animals than controls, and that was associated with increased bone fragility [34]. Magnesium may affect bone quality, independent of bone density. Lower magnesium has been associated with increased crystal size and reduced bone mass, that contribute to osteoporosis. Magnesium levels may supplement the ability of bone mineral densities to predict fractures. Consistent with these experimental studies, our study showed that lower serum magnesium levels were associated with an increased risk of fracture, and the combination of serum magnesium levels and lumbar spine BMD levels improved the prediction of fractures. Magnesium is an agonist of the calcium-sensing receptor [35]. A previous experimental study showed that magnesium could suppress PTH secretion from the parathyroid glands, particularly at moderately low extracellular calcium levels [36]. Although a lower serum magnesium level may increase the fracture risk through its effect on PTH, no significant difference in PTH values between the two magnesium groups was observed in our study.

Associations of calcium, phosphate and PTH levels with the risk of fractures were not demonstrated in our study. This could be because these factors are well-known treatment targets in the field of mineral and bone disorders, and therapeutic interventions for these factors had already been initiated in our patients. We should recognize the clinical importance of magnesium with regard to the risk of fractures, especially in patients with low lumbar spine BMD levels.

In hemodialysis patients, hypomagnesemia is commonly observed because of the complex effects of magnesium intake, drugs, and the dialysate magnesium concentration. The major factors that affect magnesium diffusion during dialysis are the concentration gradient across the dialysis membrane and the Gibbs-Donnan effect [37]. Because the dialysate magnesium concentration in Japan is 1.2 mg/dL, it is worth investigating whether high magnesium dialysates provide benefits with regard to reducing the risk of fractures.

There are some limitations of this study. First, this study was conducted in a single center and included a relatively small sample size. Second, our study had an observational retrospective design; therefore, no inferences about a causal relationship between predictive variables and fracture risk could be made. The results of our study need to be confirmed in prospective studies with larger sample sizes.

In conclusion, the combination of lower serum magnesium and low lumbar spine BMD was associated with a high risk of incident fractures in patients undergoing hemodialysis. Although magnesium has long been under recognized in the field of mineral and bone disorders in CKD patients, the combination of serum magnesium level and lumbar spine BMD may be useful for assessing the fracture risk in patients undergoing hemodialysis.

## Supporting information

S1 Dataset (XLS)
